# HIV Pre-exposure Prophylaxis Does Not Increase Gonorrhea and Chlamydia Incidence in Young Black and Hispanic Men who Have Sex With Men: An Observational Cohort Study

**DOI:** 10.1093/ofid/ofaf605

**Published:** 2025-11-07

**Authors:** Octavio C Mesner, Rishabh Jain, Aditi Ramakrishnan, Derrick D Matthews, Jeremy T Goldbach

**Affiliations:** Brown School, Washington University, St Louis, Missouri, USA; Brown School, Washington University, St Louis, Missouri, USA; Division of Infectious Diseases, Department of Medicine, Washington University School of Medicine, St Louis, Missouri, USA; Health Behavior, University of North Carolina, Chapel Hill, North Carolina, USA; Brown School, Washington University, St Louis, Missouri, USA

**Keywords:** Black and Hispanic men who have sex with men, chlamydia, gonorrhea, HIV pre-exposure prophylaxis, sexually transmitted infections

## Abstract

**Background:**

HIV pre-exposure prophylaxis (PrEP) use has been linked with increases in sexually transmitted infection (STI) incidence. Despite efforts to expand PrEP uptake among young Black and Hispanic men who have sex with men (YBHMSM), little research has been done to understand the impact of PrEP on STI incidence within these communities. We examine the effect of PrEP use on gonorrhea and chlamydia (NG/CT) incidence, condom use, and external STI testing (ie, outside of study visits).

**Methods:**

In a longitudinal cohort of HIV-negative YBHMSM (ages 16–24 years), we modeled the effect of PrEP use on study-external STI testing and number of condomless sex partners during the following 6 months using mixed-effects generalized linear models. We modeled the effect of PrEP use on NG/CT incidence using time-updated proportional hazard regression.

**Results:**

While on PrEP compared with periods not on PrEP, participants reported on average 2.51 (adjusted beta; 95% CI, 1.51–3.51; *P* < .001) more condomless sex partners and were 2.28 (adjusted OR; 95% CI, 1.48–3.52; *P* < .001) times as likely to report study-external STI testing during the following 6 months. NG/CT incidence did not increase (adjusted HR, 0.75; 95% CI, 0.45–1.27; *P* = .286) while on PrEP compared with not on PrEP.

**Conclusions:**

Condomless sex increased with PrEP use; however, its potential to elevate STI acquisition or prolonged duration of infection may be mitigated by PrEP-associated routine testing. Efforts to expand PrEP uptake among YBHMSM appear unlikely to exacerbate the STI epidemic.

Human immunodeficiency virus pre-exposure prophylaxis (PrEP) is very effective at reducing HIV acquisition in various populations, such as men who have sex with men (MSM) [1–3]. However, its use has been associated with increases in bacterial sexually transmitted infections (STIs), such as gonorrhea and chlamydia (NG/CT), raising concerns about its broader implications for the STI epidemic and STI screening guidelines when on PrEP [4–10].

The disproportionate burden of HIV on young Black and Hispanic MSM underscores the importance of PrEP uptake within these communities as a key pillar in efforts to end the HIV epidemic [11–15]. Despite this urgency, there remains a notable gap in research examining the specific impact of PrEP on STI incidence within these communities, likely due to limited longitudinal cohort data.

Studying the relationship between PrEP and STIs presents several methodological challenges. Increased STI screening among PrEP users can lead to higher detection rates, introducing potential screening bias that may inflate estimates of STI incidence [16]. Additionally, faster treatment of STIs among PrEP users may reduce the duration of infectiousness but could also increase time at risk for new infections [17]. Disentangling causal effects of PrEP from noncausal associations is another challenge. For instance, individuals with greater numbers of condomless sex partners may be more likely to initiate PrEP [18; confounding analyses of STI risk, whereas behavioral changes due to PrEP such as risk compensation—altering behaviors previously aimed at HIV and STI prevention, notably with increased condomless anal sex—would directly contribute to increased STI acquisition [5, 16, 19, 20].

While accurately quantifying STI incidence associated with PrEP is important, it provides only a partial understanding of PrEP's broader impact on population-level STI dynamics. Specifically, PrEP's integration with routine STI screening and timely treatment may shorten the duration of infectiousness for STIs, which would go undetected in the absence of regular testing. This reduction in infectious periods could significantly lower transmission rates within sexual networks, thereby decreasing overall population-level STI incidence [17].

This paper aims to evaluate the impact of PrEP use on the incidence of gonorrhea and chlamydia (NG/CT) among young Black and Hispanic MSM. Moreover, given their role mediating the impact of PrEP on STIs, this study also explores the influence of PrEP on condom use and on study-external STI testing. By using a longitudinal cohort with routine 6-month NG/CT testing, this study avoids some of the challenges mentioned above.

## METHODS

### Study Population

The Healthy Young Men's Cohort Study (HYM) is a longitudinal cohort of young Black, Hispanic, and Black/Hispanic multiracial/ethnic sexual minority men. Enrollment for HYM ran from May 2016 through September 2017 and resumed in October 2021. The eligibility criteria for HYM are the following: (a) age 16 to 24 years; (b) assigned male sex at birth; (c) identify as gay, bisexual, or uncertain sexual orientation; (d) identify as Black and/or Latino/Hispanic (abbreviated as Hispanic in this paper); (e) report at least 1 sexual encounter with a man within the previous 12 months; and (f) live in Los Angeles or the surrounding area [21]. Observations are conducted at 6-month intervals, where participants respond to a survey questionnaire and undergo HIV/STI and drug testing. To be included in this analysis, participants must have at least 1 negative urine/rectal NG/CT nucleic acid amplification test (NAAT) followed by another within 15 months, must not have had HIV at the time of the first negative NG/CT test, and must have responded to survey questions on PrEP use.

### Patient Consent

All participants provided written, informed consent in accordance with federal and institutional guidelines. The Children's Hospital of Los Angeles Institutional Review Board (CHLA # CHLA-14-00279) reviewed and approved the study protocol and consent process before initiation.

### Data and Definitions

The study survey collects PrEP use information in 2 questions. Participants are first asked “Have you used any PrEP in the past 6 months to reduce your risk of getting HIV?” If a participant endorses the item, only then are they asked about their current PrEP use. The initial survey assessed current PrEP use by asking “Are you currently taking any pre-exposure prophylaxis (PrEP) medication, such as Truvada, to reduce your risk of HIV transmission?” with a yes, no, or decline response. Later the question was changed to “What types of PrEP are you currently using to reduce the risk of getting HIV?” where participants checked all boxes that applied in order to reflect that new drugs gained Food and Drug Administration approval for PrEP after the study began. For consistency, our inferential models do not distinguish between types of PrEP. We consider a participant to be on PrEP at the date of their study visit if they indicate current PrEP use of any form; otherwise, we consider a participant to not currently use PrEP. The value is coded as missing if a participant declines or does not respond.

To assess condom use, participants are asked, “In the last 6 months, with how many people did you have insertive or receptive sex when you did not use a condom? If you always used a condom, enter 0.” For external STI testing, done outside of study visits, participants are asked, “Before today, have you been tested for a sexually transmitted disease or infection (STD/STI) in the last 6 months?” We use these responses directly.

Participants were routinely screened for urine and rectal gonorrhea and chlamydia regardless of symptom status using nucleic acid amplification tests at the visit on site or at partnering test sites. In a small number of instances, the study accepted documented, external test results performed within 2 weeks of the study visit. To assess combined NG/CT incidence, we constructed a composite test result for each participant visit by combining the results from these 4 tests to be positive if any of the 4 were positive and negative if all tests were negative. Screening for HIV and syphilis are done together with NG/CT tests. Participants who tested positive for any were referred to an HIV and STI clinic and confirmatory testing and treated according to the Centers for Disease Control and Prevention and Los Angeles County guidelines.

Information on race, financial stability, education level, methamphetamine use, and health care coverage also came from survey responses and could change over time. Age at time of visit was calculated based on participant date of birth and visit date. Prior NG/CT infection was defined as ever having a positive urine and/or rectal NG/CT composite before visit. Financial stability was a scale (1 to 6) corresponding to how often participants reported running out of money for basic needs in the past 6 months; 1 corresponds to not at all, 2 to less than once per month, 3 once a month, up to 6 corresponding to many times per week.

Like many longitudinal studies run from 2020 to 2021, the coronavirus disease 2019 (COVID-19) epidemic disrupted HYM data collection. As a result, survey and/or testing data were either missing at significant rates or missing entirely for part of 2020 and 2021. To compensate, the analysis ends all follow-up in late 2020 and allows follow-up to begin again at or after late 2021 if/when a participant has a negative composite test.

### Statistical Modeling

To evaluate the effect of PrEP on NG/CT incidence, the analysis begins follow-up for each participant at baseline, defined as a participant's first negative NG/CT composite result. Follow-up ends at either a subsequent positive composite result or at loss to follow-up, defined as no subsequent testing within 1.25 years of the last NG/CT test or HIV acquisition. Participant follow-up may restart at a subsequent negative composite result, allowing participants to contribute multiple follow-up periods and incident cases. As participant follow-up progresses, current PrEP use and other factors may change over time. As such, a participant's follow-up may span several rows in the data; each row aligns current PrEP use and other factors at the time of the visit with the duration to the subsequent composite result along with the result itself. We used a time-updated covariate, proportional hazards model clustered by participant with the data described above to quantify the effect of PrEP use on NG/CT incidence.

To evaluate the effect of PrEP on condomless sex, the analysis aligns a participant's current PrEP use and other factors taken at a visit with the number of condomless sex partners during the following 6-month period, using the subsequent visit survey, as the model outcome. Because participants are followed over time, the longitudinal data may include multiple observations/rows from several time points from the same individual. As such, current PrEP use, controlling factors, and number of condomless sex partners can change over a participant's follow-up. We used a linear mixed-effects model to account for the repeated measures from the same participants.

Evaluating the effect of PrEP use on study-external STI testing, we take a similar approach. The analysis aligns PrEP use and controlling factors taken at a visit with the yes or no response for any STI testing at the following visit for that participant. Again, participants may contribute multiple observations/rows to the analysis. We use a logistic mixed-effects model.

For all models, PrEP use and the outcome may be confounded. That is, if a factor influences PrEP use and the outcome (eg, future condom use), then an unadjusted association estimate between current PrEP use and future condom use will add the direct causal effect and the noncausal associations from each common cause. This analysis attempts to isolate the direct causal effect. Our models control for factors that could potentially influence PrEP use, from a clinical point of view. For this reason, all models include the same covariates despite possibly having different confounders for the different outcomes. Influencing current PrEP use is necessary but not sufficient for a factor to be a confounder, so by controlling for all factors that may influence current PrEP use, we will isolate an estimate of the causal effect, assuming linear associations. Moreover, with sufficient data, controlling for additional nonconfounding factors will not alter an association estimate between an exposure and outcome beyond some small error. This analysis a priori controls for age, race/ethnicity, financial stability, level of education, number of condomless partners in the past 6 months, any external STI testing in the past 6 months, any prior test record of NG/CT infection, health care coverage in the past 6 months, methamphetamine use in the past 6 months, and pre/post-COVID period of follow-up.

## RESULTS

**Figure 1.**
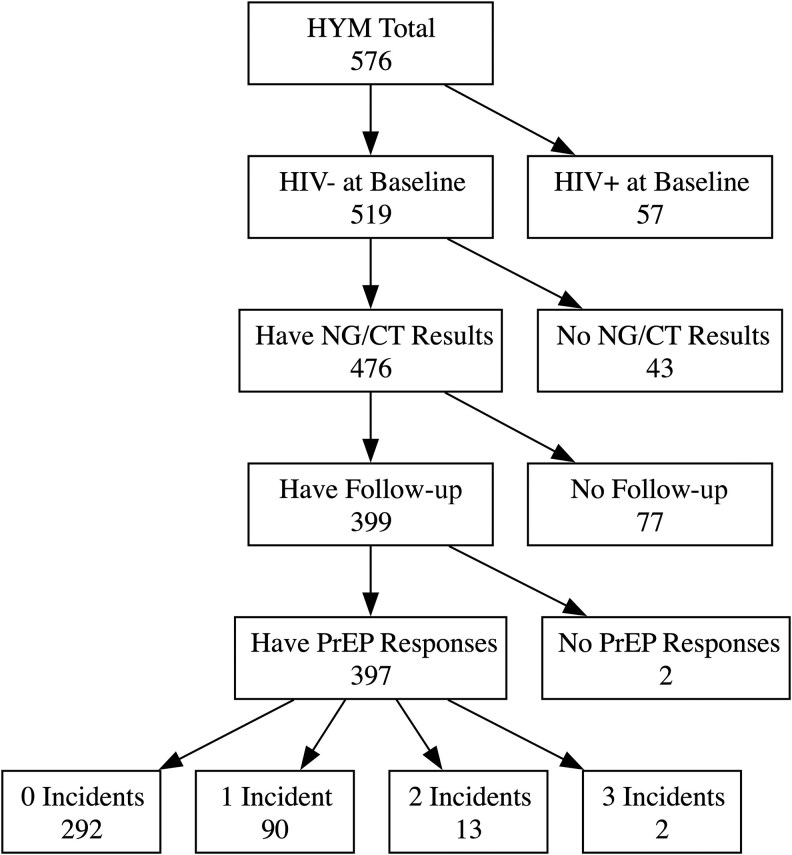
Flowchart of participants chosen for analysis. Abbreviations: NG/CT, gonorrhea and chlamydia; PrEP, pre-exposure prophylaxis.

Of the 576 participants enrolled in HYM, 397 met inclusion criteria for the analysis, with 292 participants contributing follow-up but no incident cases, 90 contributing 1 incident case, and 15 contributing >1 incident case with a median follow-up period of 2.32 years. Figure 1 shows the number of participants excluded from analysis by criteria. At baseline, those included were 64% Hispanic, 19% Black, and 18% multiracial/ethnic Black and Hispanic or other race, with a mean age (SD) of 22.95 (2.18) years and 8 (2%) participants under 18.

**Figure 2.**
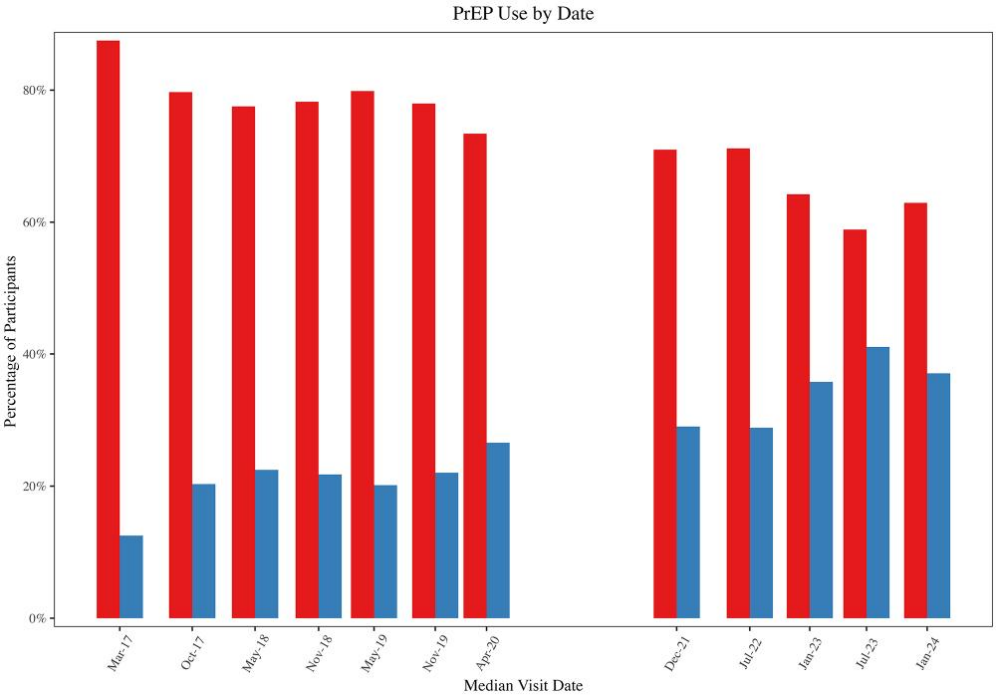
This figure gives the proportion of participants on PrEP (in blue) and not on PrEP (in red) by date, showing an upward trend in PrEP use over time. Abbreviation: PrEP, pre-exposure prophylaxis.

Sixty-seven participants were on PrEP at baseline and 330 were not, though PrEP uptake increased over time as a proportion of the cohort (Figure 2). When asked about knowledge of PrEP, >90% of the cohort consistently reported knowledge of PrEP throughout the duration of the study.

Those on PrEP at baseline were older (*P* = .004), were more educated (*P* = .002), had more condomless sex partners within the past 6 months (*P* < .001), and were more likely to be tested for an STI within the past 6 months (*P* = .003) than those not on PrEP at baseline. There were no differences by race, gender identity, financial need, health care coverage, or prior NG/CT infections. Table 1 contains a detailed summary of cohort baseline characteristics.

**Table 1.** Baseline Characteristics by PrEP Status

		PrEP Status at Baseline		
Variable	Total (n = 397)	Not on PrEP (n = 330)	On PrEP (n = 67)	*P* Value
Age at baseline				.004
Mean (SD), y	22.95 (2.17)	22.80 (2.18)	23.72 (1.98)	
Race				.200
Hispanic	252 (63.64)	203 (61.70)	49 (73.13)	
Black	74 (18.69)	63 (19.15)	11 (16.42)	
Multiracial/ethnic	70 (17.68)	63 (19.15)	7 (10.45)	
Financial stability (past 6 mo)				.400
Scale, mean (SD)	2.17 (1.40)	2.18 (1.39)	2.12 (1.49)	
Education				.002
High school or less	103 (26.08)	92 (28.05)	11 (16.42)	
Some college/vocational	192 (48.61)	164 (50.00)	28 (41.79)	
Bachelor’s or more	100 (25.32)	72 (21.95)	28 (41.79)	
Health care coverage (past 6 mo)				.073
Consistent coverage	276 (73.60)	227 (71.84)	49 (83.05)	
None/inconsistent coverage	99 (26.40)	89 (28.16)	10 (16.95)	
Condomless sex (past 6 mo)				<.001
No. of partners, mean (SD)	4.17 (8.59)	3.26 (7.57)	8.20 (11.37)	
Any STI testing (past 6 mo)				.003
No	67 (16.96)	64 (19.51)	3 (4.48)	
Yes	328 (83.04)	264 (80.49)	64 (95.52)	
Prior NG/CT infection				.11
No	344 (86.65)	290 (87.88)	54 (80.60)	
Yes	53 (13.35)	40 (12.12)	13 (19.40)	
Period				.002
Pre-COVID	339 (85.39)	290 (87.88)	49 (73.13)	
Post-COVID	58 (14.61)	40 (12.12)	18 (26.87)	
Methamphetamine use (6 mo)				.100
No	334 (92.39)	279 (93.94)	55 (87.30)	
Yes	26 (7.22)	18 (6.06)	8 (12.70)	

Data are presented as No. (%) unless otherwise indicated. *P* values were generated with the Wilcoxon test for numeric factors and the chi-square or Fisher exact test for categorical.

Abbreviations: COVID, coronavirus; NG/CT, gonorrhea and chlamydia; PrEP, pre-exposure prophylaxis.

Seventeen participants were confirmed to have seroconverted to HIV-positive status during follow-up, after which they were considered lost to follow-up. Of those, 10 consistently reported no PrEP use throughout their follow-up duration. The other 7 all reported no current PrEP use at or immediately before seroconversion. Of these, 5 reported minimal or intermittent PrEP use while 2 reported consistent PrEP use for a period but indicated no PrEP use before seroconversion.

### Statistical Models

As discussed, control variables were all chosen a priori; however, we did not include methamphetamine use due to small numbers at baseline. We were able to include all other variables discussed in the Methods. Table 2 contains parameter point estimates, 95% CIs, and *P* values for covariates in all 3 models.

**Table 2.** Regression Results

	Condomless Sex Partners		External STI Testing		NG/CT Acquisition	
Factor	Beta (95% CI)	*P* Value	OR (95% CI)	*P* Value	HR (95% CI)	*P* Value
On PrEP at visit						
No	0.0		1.0		1.0	
Yes	**2.51 (1.51 to 3.51)**	**<.001**	**2.28 (1.48 to 3.52)**	**<.001**	0.75 (0.45 to 1.27)	.286
Age at visit						
Per 1-y increase	0.00 (−0.21 to 0.22)	.965	0.96 (0.89 to 1.04)	.350	**0.91 (0.83 to 0.99)**	**.021**
Race/ethnicity						
Hispanic	0.0		1.0		1.0	
Black	−0.66 (−1.87 to 0.54)	.278	0.94 (0.61 to 1.44)	.763	1.14 (0.70 to 1.88)	.598
Multiracial/ethnic	−0.92 (−2.13 to 0.29)	.136	1.40 (0.83 to 2.23)	.158	0.83 (0.49 to 1.43)	.506
Financial stability at visit						
Per scale unit	0.17 (−0.15 to 0.49)	.288	0.93 (0.83 to 1.04)	.209	0.97 (0.84 to 1.12)	.694
Education						
High school or less	0.0		1.0		1.0	
Some college/vocational	0.04 (−1.09 to 1.18)	.939	1.07 (0.71 to 1.60)	.759	0.85 (0.55 to 1.31)	.458
Bachelor’s or more	0.85 (−0.48 to 2.19)	.208	1.02 (0.63 to 1.64)	.948	0.75 (0.44 to 1.27)	.281
Condomless sex (past 6 mo)						
Per partner	**0.50 (0.45 to 0.54)**	**<.001**	1.02 (1.00 to 1.05)	.078	1.01 (1.00 to 1.03)	.105
Any STI testing (past 6 mo)						
No	0.0		1.0		1.0	
Yes	0.40 (−0.63 to 1.43)	.445	**1.63 (1.10 to 2.40)**	**.014**	1.12 (0.69 to 1.83)	.644
Prior NG/CT infection at visit						
No	0.0		1.0		1.0	
Yes	0.34 (−0.67 to 1.35)	.511	**0.67 (0.47 to 0.97)**	**.033**	**2.09 (1.26 to 3.45)**	**.004**
Health care (past 6 mo)						
Consistent	0.0		1.0		1.0	
None/inconsistent	−0.22 (−1.10 to 0.66)	.628	0.78 (0.57 to 1.07)	.120	1.26 (0.87 to 1.81)	.221
Period						
Pre-COVID	0.0		1.0		1.0	
Post-COVID	−0.63 (−1.90 to 0.64)	.329	1.07 (0.67 to 1.70)	.780	0.79 (0.41 to 1.54)	.492

Bold values are significant at *P* < 0.05.

Abbreviations: COVID, coronavirus disease; HR, hazard ratio; NG/CT, gonorrhea and chlamydia; OR, odds ratio; PrEP, pre-exposure prophylaxis; STI, sexually transmitted infection.

While on PrEP, participants reported on average 2.51 (beta; 95% CI, 1.51–3.51; *P* < .001) more condomless sex partners during the following 6 months compared with when not on PrEP, controlling for potential confounders. Moreover, for each condomless sex partner during the prior 6 months, participants reported an additional 0.50 (beta; 95% CI, 0.45–0.54; *P* < .001) condomless sex partners during the following 6 months. There were no statistically significant differences by age, race, financial stability, level of education, prior STI testing, prior NG/CT infection, or health care coverage, after controlling for other factors.

While on PrEP, participants were 2.28 (OR; 95% CI, 1.48–3.52; *P* < .001) times as likely to report having been tested for an STI during the following 6 months, compared with when not on PrEP, controlling for potential confounders. Reporting having been tested for an STI compared with not during the past 6 months, participants were 1.63 (OR; 95% CI, 1.10–2.40; *P* = .014) times as likely to report having been tested for an STI during the following 6 months. When our study had a prior record of NG/CT infection compared with no record, participants were less likely (OR, 0.67; 95% CI, 0.47–0.97; *P* = .033) to report being tested during the following 6 months. Race, age, financial stability, level of education, health care coverage, and period were not significantly associated.

**Figure 3.**
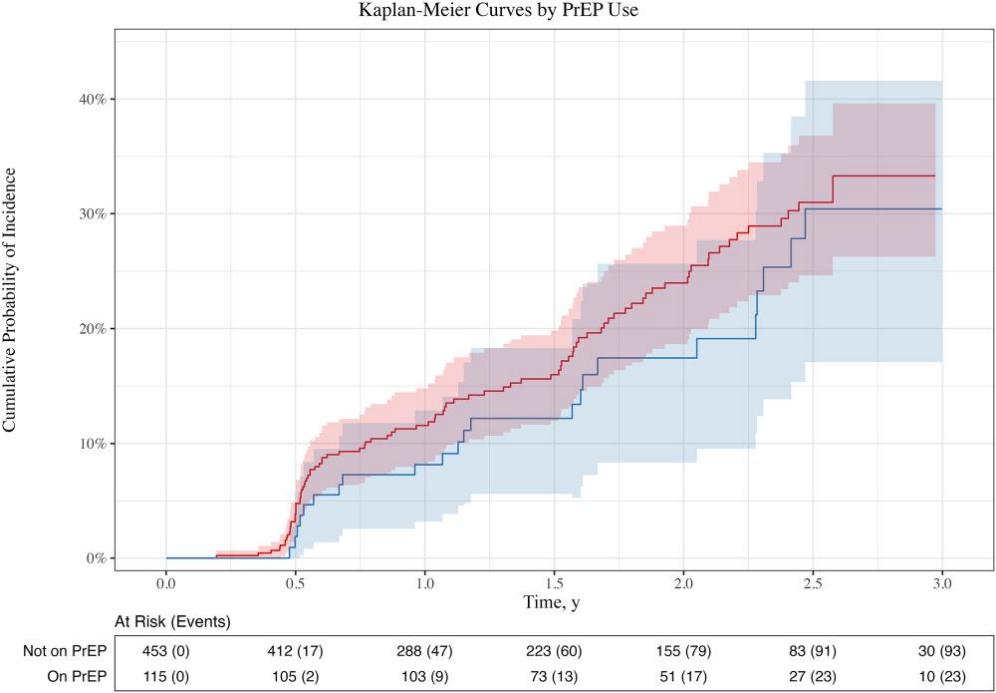
This figure provides Kaplan-Meier curves, illustrating the cumulative probability of incidence over follow-up by PrEP use. The red curve represents follow-up not on PrEP and the blue curve corresponding to follow-up on PrEP. Abbreviation: PrEP, pre-exposure prophylaxis.

The Kaplan-Meier curves (Figure 3) show unadjusted cumulative probabilities of NG/CT acquisition over time by PrEP use. Due to time-updated PrEP status and the possibility of >1 follow-up period per participant, there are nuances to this graph that bear further clarification. Participants who change PrEP status will contribute follow-up time to the risk group corresponding to their current PrEP status; thus, a single participant may contribute to both curves but at different periods of their follow-up. Because participants can contribute additional follow-up again after an event or censor, the number at risk at any given time corresponds to the number of follow-up periods contributed in total, which may include the same participant more than once. Due to software limitation, the confidence bands shown for each curve are generated assuming independent observations, which is not the case. Our confidence bands should be wider than what is shown here to account for the potential dependence between observations from the same individuals. However, for these curves, even with conservative confidence bands, there is significant overlap, indicating no statistical difference in acquisitions over time by PrEP status.

The NG/CT-incident model satisfied the proportional hazards assumptions. PrEP use was not associated with NG/CT incidence (HR, 0.75; 95% CI, 0.45–1.27; *P* = .286). For each additional year of age, acquisition decreased by 9% (HR, 0.91; 95% CI, 0.83–0.99; *P* = .021). When our study had a prior record of NG/CT infection compared with not, acquisition increased by a factor of 2.09 (95% CI, 1.26–3.45; *P* = .004). No other factors were significantly associated with NG/CT incidence. An unadjusted proportional hazards model, including only PrEP use, yielded similar results (HR, 0.81; 95% CI, 0.51–1.30; *P* = .379).

## DISCUSSION

In this cohort of young Black and Hispanic MSM living in Los Angeles, California, we observed that while on PrEP participants reported a greater number of condomless sex partners and more frequent STI testing. Importantly, despite changes in sexual behavior, there was no increased incidence of NG/CT when participants were on PrEP. By utilizing a longitudinal, observational cohort with routine 6-month NG/CT testing, this study offers several advantages. First, it minimizes screening bias with consistent testing regardless of PrEP status. Second, the longitudinal study design enables control of prior sexual behaviors and other potential confounding factors. Third, the use of primarily nonclinical NG/CT tests enhances the study's ability to evaluate the real-world effects of PrEP, including its integration with routine screening and timely treatment along with potential risk compensation, within this population.

Considering factors that may influence a participant's number of condomless sex partners, the analysis suggests that an average increase of 2.5 condomless sex partners over a 6-month period is associated with PrEP use within this population. This finding affirms existing research suggesting that PrEP use may lead to risk compensation, specifically a reduction in condom use [4–10]. The model also suggests that having more prior condomless sex partners predicts more future condomless sex partners as well. Further, only PrEP use and past condom use were strong predictors of future condom use, indicating that these factors, rather than demographic characteristics such as age, race, or education level, etc., are more influential in shaping this behavior.

Moving to study-external STI testing, STI screening outside of study visits was 2.3 times more likely over a 6-month period when on PrEP, likely due, at least in part, to Centers for Disease Control and Prevention (CDC) STI screening guidelines. For the duration of this study, the CDC has recommended quarterly HIV testing and joint bacterial STI testing, currently depending on exposure [22]. It may also be the case that PrEP use captures other behaviors that make STI acquisition more likely, consequently prompting participants to seek more testing. Considering other factors, participants were 63% more likely to undergo study-external testing when having reported study-external testing during the prior 6 months but 33% less likely when they had had a prior NG/CT infection. No other factors were significantly associated with study-external testing, including a participant's prior number of condomless sex partners. It is possible that some participants may have relied on the study for their testing, and having testing provided may have reduced the need for study external testing. Only 17% of participants at baseline reported no study-external testing, however, making it a small minority that relied exclusively on the study for their testing. While study-external testing itself only makes sense within the context of study participants, we would still expect this model to reflect STI testing patterns in the larger population if being in this study does not differentially affect study-external testing by group, which is admittedly difficult to determine.

Finally, we look at NG/CT incidence. Participant risk of NG/CT acquisition decreased by 9% per year as participants aged and doubled with prior positive NG/CT results, again consistent with other research [8]. No other factors were associated with NG/CT acquisition after controlling for age and prior NG/CT infection, including PrEP use. Moreover, the lack of evidence supporting PrEP use leading to increased NG/CT incidence in the adjusted proportional hazards model remained consistent with both the unadjusted proportional hazards model and the Kaplan-Meier curves and may indicate minimal confounding from control variables. Further, the model suggests that sexual networks may predict NG/CT incidence better than sexual behavior. Prior NG/CT infection, a positive predictor of acquisition, may be an indicator of sexual network, whereas a participant's number of condomless sex partners was not as helpful for prediction.

Several studies provided PrEP to all participants along with quarterly or biannual HIV/STI screening [5–8]. These studies are well positioned to capture increases in STI incidence and sexual risk after PrEP but may not be well suited to capture longer-term effects of PrEP use, which frequently include increased testing and treatment. In contrast, our study conducted biannual STI screening regardless of PrEP status and only included external STI test results performed within 2 weeks of study visits. This design may have limited our ability to detect short-lived NG/CT infections; however, it does position this analysis to assess the combined effect of PrEP use and its corresponding testing on longer-term infection rates. Thus, while our findings may appear to diverge from studies focused on immediate, post-PrEP STI increases, they offer insight into the overall impact of associated testing practices on NG/CT risk over time. Further, these findings suggest that the increased frequency of STI testing associated with PrEP use likely mitigates the impact of the increased condomless sex partners on NG/CT acquisition. Increased testing could lead to earlier diagnosis and treatment, thereby limiting the duration of infectiousness and preventing transmission.

Though this analysis primarily focuses on NG/CT acquisitions, it is important to address the 17 HIV seroconversions that occurred during follow-up. Given the efficacy of PrEP for preventing HIV, it seems likely that consistent PrEP use could have prevented most if not all of these seroconversions. This underscores the importance of engaging in efforts to understand and tackle barriers to consistent PrEP access and uptake among diverse populations.

### Limitations and Generalizability

This study has several limitations. First, PrEP use, number of condomless partners, study-external STI testing, and other information are self-reported and may include some error, possibly due to recall bias, social desirability bias, or misunderstanding of questions. PrEP adherence may vary by participant and over time. Recall may impact responses to questions on the number of condomless partners but may have a smaller impact on current PrEP use and external testing. The study uses computer-based questionnaire submission to reduce social desirability bias. At each visit, participants are encouraged to ask a research assistant if they do not understand a question. Because we have no reason to suspect differential bias by PrEP use, these errors would likely contribute noise without changing the overall signal, increasing standard errors but not changing point estimates, such as betas, odds ratios, or hazard ratios. It is also important to acknowledge that the number of condomless sex partners is an incomplete measure of STI transmission risk as it is also driven by the number of condomless events and the sexual networks of partners.

Second, our cohort is entirely from the Los Angeles region and is not representative of young Black and Hispanic MSM in other regions of the United States. Of note, the racial/ethnic composition of this cohort does not mirror those in other parts of the United States, and PrEP may be more accessible to those in regions such as the West Coast [23]. Despite these differences, our analysis and study design attempt to make the results shown here with respect to the impact of PrEP use on condom use, STI testing, and NG/CT incidence approximate those throughout the United States among young Black and Hispanic MSM who do not use methamphetamines in 3 primary ways. First, sampling bias is unlikely to influence these results; that is, if PrEP use and outcome both influenced sampling, this would alter any association estimate between PrEP and an outcome. For this cohort, PrEP use has no bearing on inclusion and seems very unlikely to influence continued participation in the study, likely rendering any sampling bias very minimal. Second, by controlling for race, age, and other factors that could influence PrEP use, we attempt to remove any linear effects these may have on association estimates between PrEP and each outcome. However, information regarding the number of sex partners with HIV was not collected consistently enough to include as a control covariate. Third, by aligning current PrEP use and other covariates taken at a given study visit with outcomes during the following 6-month period in our modeling, we eliminate any possibility that association estimates include effects of outcomes on PrEP use and covariates.

Third, our survey does not collect information about doxycycline postexposure prophylaxis (doxy PEP), which could reduce NG/CT acquisitions for more recent waves of data [24]. However, its inclusion as a covariate would depend on its role as a confounder vs mediator between PrEP use and NG/CT incidence, which bears further investigation. If doxy PEP were a mediator (ie, PrEP use influences doxy PEP use), its inclusion as a covariate in a model predicting NG/CT incidence could block the causal pathway from PrEP use to incidence; thus the association estimate for PrEP would no longer represent the total effect.

## CONCLUSIONS

This study illustrates that PrEP use is associated with increased condomless sex; however, its association with increased STI acquisition or prolonged infection durations may be mitigated by routine testing associated with PrEP use. Successful efforts to expand PrEP uptake among young Black and Hispanic MSM should decrease new HIV infections and appear unlikely to exacerbate the STI epidemic.

## Notes

***Acknowledgments.*** We thank the participants for contributing their data, without whom this work would not be possible. We would like to dedicate this work to the memory of Jeremy Goldbach, who passed away before this work was completed.

***Author contributions.*** O.M. designed the analysis and drafted the manuscript. R.J. executed the analysis. J.G. is the study PI and grant recipient. R.J., A.R., D.M., and J.G. provided valuable feedback on the manuscript.

***Data availability.*** De-identified data are available upon request.

***Financial support.*** This study was supported by the National Institutes of Health/National Institute on Drug Abuse (grant number U01 DA03926).

***Potential conflicts of interest.*** A.R. is a recipient of the HIV Research Scholars Program Award 2024-2026. All other authors report no conflicts of interest.
